# Overlap of Bickerstaff Encephalitis and Pharyngeal-Cervical-Brachial Variant of Guillain-Barré Syndrome Following COVID-19 Infection

**DOI:** 10.7759/cureus.94218

**Published:** 2025-10-09

**Authors:** Kosuke Okuma, Kentaro Hori, Shingo Kawakami, Yoko Suzuki

**Affiliations:** 1 Department of Neurology, Japanese Red Cross Omori Hospital, Tokyo, JPN

**Keywords:** anti-gq1b antibody, bickerstaff's brainstem encephalitis (bbe), covid-19, paralytic ileus, pharyngeal-cervical-brachial (pcb) variant

## Abstract

A 36-year-old woman developed numbness in both fingers and unsteadiness around 10 days after COVID-19 infection. She was admitted to our hospital after experiencing drowsiness, dysarthria, and gait disturbance within three days. On admission, her vital signs were stable, with no signs of infection. Neurological examination revealed disturbances in consciousness, ophthalmoplegia, ataxia, pharyngeal and cervical muscle weakness, and sensory abnormalities predominantly affecting the upper extremities. Brain MRI and CSF examination revealed no abnormalities, whereas the median nerve somatosensory evoked potential indicated an intracranial conduction abnormality. Based on these findings, brainstem encephalitis of non-infectious origin was suspected, and IVIg and high-dose IV methylprednisolone therapies were initiated. Subsequent serological testing revealed the presence of serum anti-GQ1b and anti-GT1a IgG antibodies, confirming the diagnosis of Bickerstaff brainstem encephalitis (BBE) and pharyngeal-cervical-brachial variant of Guillain-Barré syndrome following COVID-19 infection. The patient responded well to immunotherapy, with rapid improvement in neurological symptoms. By day 20, the patient was able to walk independently, although mild ataxia persisted. On day 24, she was transferred to another hospital for rehabilitation. This case highlights the overlap of the BBE and PCB variants as neurological complications following COVID-19 infection. The presence of anti-ganglioside antibodies, specifically anti-GQ1b and anti-GT1a, plays a crucial role in diagnosis, even when triggered by COVID-19. Early recognition and prompt immunotherapy may contribute to a favorable prognosis.

## Introduction

Bickerstaff brainstem encephalitis (BBE) is a rare variant of Guillain-Barré syndrome (GBS), characterized by the triad of altered consciousness, ataxia, and external ophthalmoplegia [1,2]. Another atypical subtype is the pharyngeal-cervical-brachial (PCB) variant, which presents with predominant weakness in the pharyngeal, neck, and upper limb muscles [3]. Both subtypes are often preceded by upper respiratory tract infections. Clinical and immunological studies have suggested overlapping features between BBE and PCB, and antiganglioside antibodies such as anti-GQ1b are frequently detected, indicating a shared autoimmune basis [1-3]. COVID-19, caused by SARS-CoV-2, is associated with various neurological complications, and its relationship with GBS is well recognized. It has been pointed out that antiganglioside antibodies are less often detected [4]. Reports of BBE following COVID-19 are limited [5-8], and to date, no cases of co-occurrence of these two variants have been documented, and their pathophysiology is unclear. This study reports a rare case of overlap of BBE and PCB variants following COVID-19 infection. Notably, prompt immunotherapy led to favorable outcomes, despite the development of paralytic ileus during the clinical course.

## Case presentation

A 36-year-old woman with a history of depression and ulcerative colitis, for which she had been treated with mesalazine suppository, developed upper respiratory symptoms around 10 days before admission. She was diagnosed with COVID-19 based on a positive SARS-CoV-2 antigen test seven days before admission. Three days before admission, she experienced numbness and unsteadiness in her hands, followed by slurred speech and gait disturbance the following day. Because of dysphagia and altered consciousness, she was admitted to a local hospital the day before and was transferred to our hospital the following day for further evaluation and treatment.

**Figure 1:**
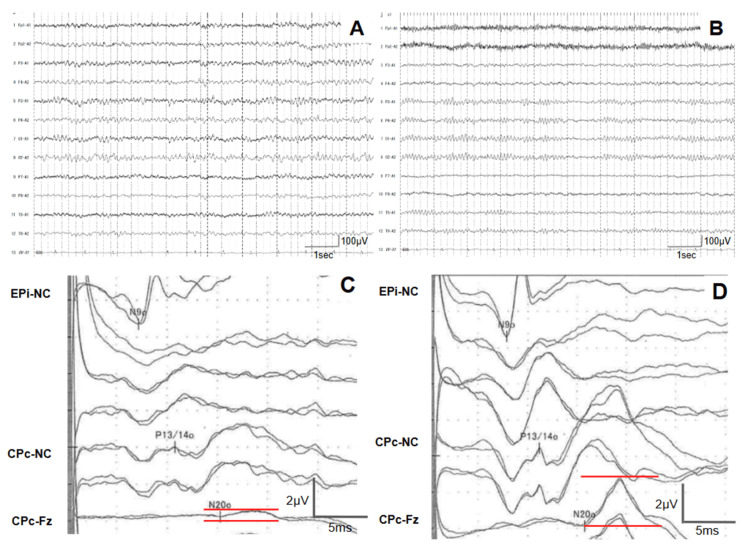
Neurophysiological examination EEG on days 2 (A) and 16 (B); SEP of the median nerve on days 2 (C) and 23 (D) (A) EEG on admission showed background activity of 8-9 Hz with a poorly organized posterior dominant rhythm, considered slow for age. (B) EEG normalized following improvement in arousal after treatment. (C) The initial SEP showed a reduction in N20 amplitude (red line). (D) Follow-up SEP after treatment demonstrated recovery of the N20 amplitude. EEG filter settings: high-cut filter, 60 Hz; time constant, 0.3 s; AC filter, on CPc, Centroparietal electrode contralateral to the stimulation; EEG, Electroencephalography; EPi, Erb’s point electrode ipsilateral to the stimulation; Fz, Midline frontal electrode; NC, Non-cephalic reference; SEP, Somatosensory evoked potential

**Figure 2:**
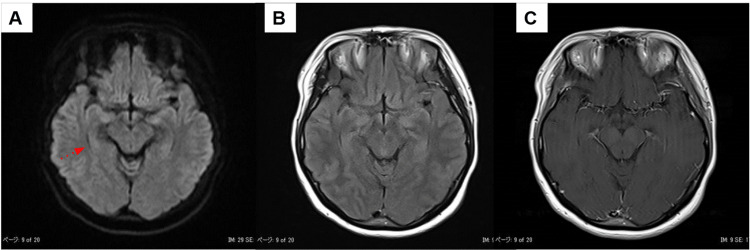
Brain MRI on admission (A) Axial DWI of the midbrain level. (B) Axial FLAIR image. (C) Axial T1-weighted image with gadolinium contrast. No intracranial hyperintense lesions or abnormal contrast enhancement were observed, including within the limbic system. DWI, Diffusion-weighted imaging; FLAIR, Fluid-attenuated inversion recovery; MRI, Magnetic resonance imaging

On admission, her vital signs were as follows: heart rate, 72 bpm; blood pressure, 104/74 mmHg; temperature, 36.5°C; SpO₂, 98% (on room air). Cardiac and respiratory sounds were normal, and no rashes were observed. Neurological examination revealed decreased consciousness (Glasgow Coma Scale score of E3V4M6). The patient was responsive to verbal stimuli but exhibited a marked nasal voice. Bilateral ptosis was observed, with pupils measuring 6 mm, sluggish light reflexes, restricted ocular movements, and a fixed central gaze. Facial muscle strength was preserved, but she tended to show involuntary crying-like expressions. Manual muscle testing revealed weakness in the neck and upper limbs (grade 4) and mild weakness in the lower limbs (grade 4-5). Sensory examination revealed dysesthesia in the bilateral upper limbs, upper trunk, and distal lower limbs and impaired vibratory sensation. Severe limb and truncal ataxia prevented sitting without support. Deep tendon reflexes were reduced or absent in all extremities, and bilateral Babinski and Chaddock signs were positive. Laboratory findings were as follows: WBC count, 7,500/μL; RBC count, 539 × 10^4^/μL; Hb, 15.9 g/dL; C-reactive protein, 0.55 mg/dL; ammonia, 23 μg/dL; procalcitonin, 0.0 ng/mL. Vitamin B1 was within normal range. Autoantibodies against collagen vascular diseases were absent. CSF analysis showed a clear appearance, an opening pressure of 10 cm H₂O, a normal cell count of 2/μL, and a protein level of 44 mg/dL (Table 1). Nerve conduction studies revealed no significant abnormalities. Electroencephalography (EEG) showed a posterior dominant alpha rhythm of 8-9 Hz, which was mildly slower than expected for her age and was poorly organized (Figure 1A). Median nerve somatosensory evoked potential (SEP) revealed the reduction of N20 amplitude, although the P13/14 onset latency was within normal limits (Figure 1C). Brain MRI revealed no intracranial hyperintense lesions or contrast enhancement (Figure 2). Based on these findings, BBE was the primary diagnostic consideration as a disorder presenting with polyneuropathy and brainstem dysfunction, and the patient was admitted for treatment.

**Table 1:** Laboratory data APTT, Activated partial thromboplastin time; ELISA, Enzyme-linked immunosorbent assay; HSV-DNA, Herpes simplex virus deoxyribonucleic acid; IFA, Indirect immunofluorescence assay; IgG, Immunoglobulin G; MPO-ANCA, Myeloperoxidase anti-neutrophil cytoplasmic antibody; OCB, Oligoclonal band; OD, Optical density; PR3-ANCA, Proteinase 3 anti-neutrophil cytoplasmic antibody; PT-INR, Prothrombin time-international normalized ratio; SS-A, Sjögren’s syndrome-related antigen A; SS-B, Sjögren’s syndrome-related antigen B; T3, Triiodothyronine; T4, Thyroxine; VZV-DNA, Varicella-zoster virus deoxyribonucleic acid

Laboratory test	Patient value	Normal range	Units
Complete blood count			
White blood cell	7,500	3,300-8,600	/µL
Red blood cell	539	386-492	x10⁴/µL
Hemoglobin	15.9	11.6-14.8	g/dL
Hematocrit	48.0	35.1-44.4	%
Platelets	33.4	15.8-34.8	x10⁴/µL
Biochemistry test			
C-reactive protein	0.55	<0.3	mg/dL
Total protein	8.5	6.6-8.1	g/dL
Albumin	4.3	4.1-5.1	g/dL
Blood urea nitrogen	14.5	8.0-20.0	mg/dL
Creatinine	0.47	0.46-0.79	mg/dL
Total bilirubin	0.8	0.4-1.5	mg/dL
Aspartate aminotransferase	16	13-30	U/L
Alanine aminotransferase	10	7-23	U/L
Creatine kinase	78	41-153	U/L
Lactate dehydrogenase	167	124-222	U/L
Sodium	138	138-145	mEq/L
Potassium	4.3	3.6-4.8	mEq/L
Chloride	99	101-108	mmol/L
Glucose	101	73-109	mg/dL
Procalcitonin	0.0	<0.05	ng/mL
Free T3	2.52	2.30-4.00	pg/mL
Free T4	1.02	0.93-1.70	ng/dL
Thyroid-stimulating hormone	0.619	0.500-5.00	µU/mL
Ammonia	23	12-66	µg/dL
Vitamin B1	40	23.1-81.9	ng/mL
Coagulation test			
PT-INR	1.04		
APTT	25.9	24.0-39.0	s
D-dimer	1.2	<1.0	µg/mL
Immunologic test			
Antinuclear antibody (IFA)	<40	<40	
Rheumatic factor	1.25	<2.0	
Anti-SS-A antibody	<1.0	<10	U/mL
Anti-SS-B antibody	1.0	<10	U/mL
Anti-thyroglobulin antibody	<10.0	<19.3	U/mL
MPO-ANCA	<1.0	<3.5	U/mL
PR3-ANCA	1.4	<3.5	U/mL
Serum anti-ganglioside antibody test (ELISA)	Interpretation (OD, blank subtracted)		
IgG class anti-GM1 antibody	- (0.011)		
IgG class anti-GQ1b antibody	++ (0.889)		
IgG class anti-GT1a antibody	++ (0.940)		
IgG class anti-GT1b antibody	- (0.018)		
IgG class anti-GD1a antibody	- (0.016)		
CSF test			
Appearance：Clear, opening pressure：10 cm H₂O			
Cell count	2	0-5	/µL
Protein	44	10-40	mg/dL
Glucose	67	50-75	mg/dL
HSV-DNA	<2x10²	<2x10²	copies/mL
VZV-DNA	<2x10²	<2x10²	copies/mL
Myelin basic protein	127	0-102	pg/mL
IgG index	0.5	<0.7	
OCBs	-	-	

**Figure 3:**
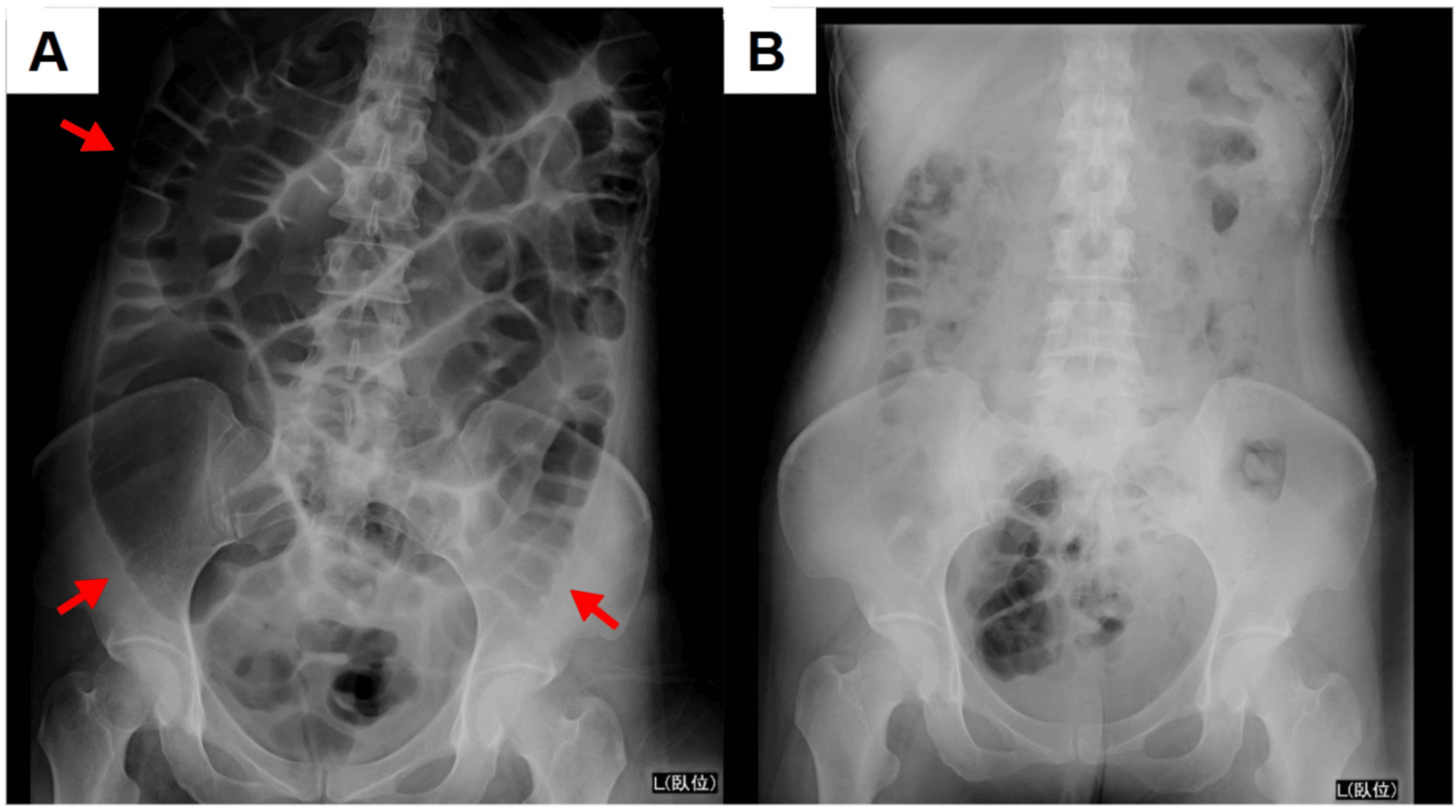
Abdominal X-rays showing paralytic ileus Radiography performed on day 4 (A) shows diffuse intestinal dilation without evidence of mechanical obstruction, which improved with conservative treatment by day 14 (B).

**Figure 4:**
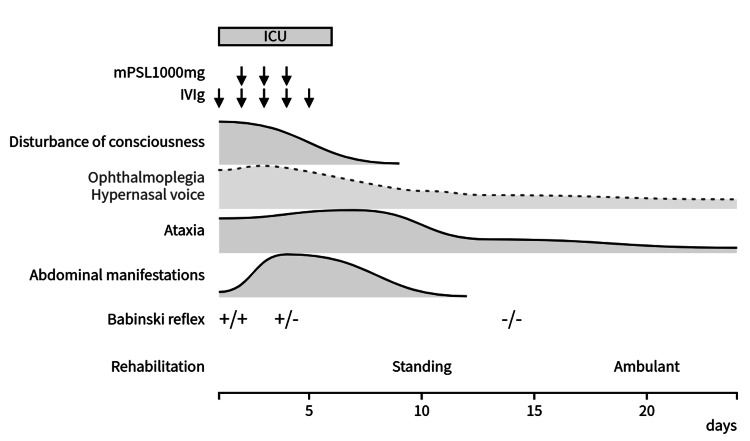
Post-admission clinical course ICU, Intensive care unit; IVIg, Intravenous immunoglobulin; mPSL, Methylprednisolone

Because of the impaired consciousness and bulbar palsy, the patient was admitted to the ICU for close monitoring. IVIg therapy (400 mg/kg/day) was administered for five days starting on the day of admission, followed by three days of methylprednisolone pulse therapy (1,000 mg/day). Her level of consciousness rapidly improved. By hospital day 8, the patient was fully alert and did not require mechanical ventilation. Subsequently, she was transferred to the general ward on day 10. Neurological symptoms improved significantly within the first week; however, ataxia became more apparent as the muscle strength recovered. Numbness in the upper limbs persisted for approximately two weeks. On hospital day 3, the patient developed abdominal distention, pain, and nausea. Abdominal radiography and CT revealed diffuse intestinal dilation without signs of obstruction, leading to the diagnosis of paralytic ileus. Fasting and central venous nutrition were initiated, and the symptoms resolved within approximately 10 days (Figure 3). A follow-up EEG demonstrated normalized background activity (Figure 1B), and SEP showed recovery of the N20 potential amplitude (Figure 1D). Serological testing was positive for serum IgG anti-GQ1b and anti-GT1a antibodies, confirming the diagnosis of BBE with features of the PCB variant. The patient regained the ability to stand by day 10 and walked unassisted by day 20. Ultimately, the patient was transferred for rehabilitation on day 24, with mild bilateral abduction limitation and lower limb-dominant ataxia at discharge (Figure 4).

## Discussion

BBE and its PCB variant are rare subtypes of GBS. Disease-specific antibodies are presumed to be anti-GQ1b antibodies against BBE and anti-GT1a antibodies against PCB variant [1-3]. Clinically, approximately 60% of BBE cases present with pharyngeal palsy, suggesting that these two conditions may lie on the clinical spectrum. This is further supported by serological findings, which show that 90% of anti-GT1a antibody-positive cases are also positive for anti-GQ1b antibodies due to cross-reactivity [3,5].

In the present case, the patient developed BBE approximately 10 days after COVID-19 infection, initially presenting with the classical triad of BBE and bilateral upper limb paresthesia. She exhibited marked nasal speech, sensory disturbances predominantly in the upper limbs and trunk, and muscle weakness. Based on these clinical features, a coexisting PCB variant was suspected, supported by the presence of serum IgG anti-GQ1b and anti-GT1a antibodies. Abnormal median nerve SEP findings, such as reduced N20 amplitude or disappearance of the N20 component with preserved P13/14 onset latency, are frequently observed in BBE and are considered useful for early diagnosis [9]. In the present case, such findings were detected by SEP two days after admission, despite the absence of any brain MRI abnormalities.

COVID-19 has been recognized as a potential trigger for GBS. A systematic review of 436 patients reported a mean age of 61 years with a male predominance, with GBS symptoms typically emerging at an average of 19 days following the onset of COVID-19. The most common clinical subtype is acute inflammatory demyelinating polyneuropathy. Among the reviewed cases, four were classified as PCB variants, whereas none were identified as BBE. A notable feature of COVID-19-associated GBS is the low detection rate of antiganglioside antibodies. Among 16 reported cases of Miller-Fisher syndrome (MFS), anti-GQ1b antibodies were detected in only two cases, a significantly lower rate than the general detection frequency of approximately 90%. This suggests that the pathogenesis of MFS differs between patients with and without COVID-19 infection [4]. Given the shared pathophysiological features between BBE and MFS [10], BBE cases occurring in the context of COVID-19 may also exhibit a lower frequency of anti-GQ1b antibody positivity.

We compared the present case with five previously reported cases of BBE following COVID-19 infection [5-8] (Table 2). The average interval between infection and onset was 12.2 days (range: 4-28 days), and most patients were positive for the anti-GQ1b antibody. All patients received IVIg therapy and steroid pulse therapy. In our case, the patient developed paralytic ileus during hospitalization. One previously reported case was complicated by takotsubo cardiomyopathy, indicating that careful monitoring for autonomic dysfunction is necessary. Although neurological assessment in the acute phase may be limited by intubation, none of the previously reported cases were considered to involve a coexisting PCB variant. Most patients recovered to a level of independent ambulation. However, one fatal case involved negative serum autoantibody results and MRI findings showing bilateral midbrain abnormalities.

**Table 2:** Review of the previously reported cases of BBE following COVID-19 infection BBE, Bickerstaff brainstem encephalitis; COVID-19, Coronavirus disease 2019; IVIg, Intravenous immunoglobulin; IVMP, Intravenous methylprednisolone; PE, Plasma exchange; PO-PSL, Per-oral prednisolone

Literature	Age/sex	Onset interval	Initial symptoms	Complications	Serum antibodies	Treatment	Outcome
Llorente Ayuso L, et al. [5]	72 F	28 days	Dizziness	–	GD1a	IVMP, followed by PO-PSL	Ambulatory
Kimura M, et al. [6]	68 F	2 weeks	Dysarthria, gait disturbance	Takotsubo cardiomyopathy	GQ1b, GT1a, GM1/GT1a	IVIg, IVMP, PE	Ambulatory
Mori D, et al. [7] (case 1)	51 M	4 days	Disturbance of consciousness	–	GQ1b	IVIg, IVMP	Ambulatory
Mori D, et al. [7] (case 2)	28 F	Unknown	Disturbance of consciousness	Septic shock	Negative	IVIg, IVMP, PE	Death
Naoki I, et al. [8]	28 F	5 days	Paresthesia, limb weakness, unsteadiness	–	GQ1b	IVIg, IVMP, PE	Ambulatory
Present case	36 F	10 days	Paresthesia in both hands	Paralytic ileus	GQ1b, GT1a	IVIg, IVMP	Ambulatory

In general, patients who are negative for anti-GQ1b antibodies tend to exhibit abnormal brain MRI findings, increased CSF cell counts and protein levels, and prolonged disturbances of consciousness [11]. In the present case, although BBE developed following COVID-19 infection, brain MRI and CSF findings were both normal. Consciousness and cranial nerve symptoms improved significantly within approximately 1 week. The subsequent detection of anti-GQ1b antibodies was consistent with the typical features of antibody-positive cases. These findings suggest that even when BBE is triggered by COVID-19, testing for anti-ganglioside antibodies remains important for diagnosis.

## Conclusions

This study presents a case of overlap of BBE and PCB variants following COVID-19, with a favorable outcome obtained through prompt immunotherapy. In patients presenting with an acute onset of impaired consciousness and brainstem symptoms after COVID-19, BBE should be considered in the differential diagnosis. When symptoms extend beyond the classical triad, the possibility of a coexisting variant, such as the PCB variant, warrants consideration. Assessment of anti-ganglioside antibodies remains essential for diagnosis, even in cases triggered by COVID-19.
